# *Zea maize* reference materials for genetically modified organism detection in Mexico

**DOI:** 10.1002/ece3.5667

**Published:** 2019-10-02

**Authors:** Esther Castro Galvan, Mauricio Maldonado Torres, Melina Pérez Urquiza

**Affiliations:** ^1^ Materials Metrology Area Centro Nacional de Metrología (CENAM) Querétaro México

## 1 INTRODUCTION

Maize is one of the most important crops in the world. Currently, Mexico is the 7th largest producer in the world behind Brazil and the European Union (World Atlas, 2019). In Mexico, maize is the most important crop because it is a basic component of the Mexican diet. Maize has been used since the original settlers in Mexico domesticated a wild plant known as *teocintle* around 9,000–10,000 years ago (Kato, Mapes, Mera, Serratos, & Bye, 2009). Even if some authors still discuss its origin, there is no doubt of its importance in the world (Kato et al., 2009; Matsuoka et al., 2002; CONABIO, 2012).

The recombinant DNA technique was a revolutionary discovery during the development of genetic engineering because it was the beginning of genetically modified organisms (GMOs), whose commercialization started around 1996 (Pellegrini, 2011). In Mexico, during the period of 1998–2000, there was a discussion related to the presence of GMO in Mexico, producing an intense public debate. Because of the fact that there are different, traditional varieties in Mexico, a group of scientists considered that they could be in danger because of GMO cross‐contamination (CONABIO, 2019).

Several researchers have performed analyses on Mexican Maize looking for GMO, mainly in the Oaxaca region (Agapito Tenfen & Wickinson, 2018; Matsuoka et al., 2002; Ortíz‐García et al., 2005a; Pearce, 2002; Piñeyro‐Nelson et al., 2009). Nevertheless, no one agrees with the results and there are still controversies around the conclusions of these studies. Quist and Chapela concluded that there was GMO contamination, specifically in the Oaxaca region (Davis & Ignacio, 2001). However, in 2005, Ortíz‐García et al. (2005a, 2005b) declared the absence of GMO in the same Oaxaca region. In a more recent publication, Piñeyro‐Nelson et al. (2009) concluded again that GMOs were present in landrace populations and suggested the need of a sampling plan and monitoring protocols, in order to achieve reliable results.

Agapito Tenfen and Wickinson (2018) make a review trying to identify the main technical problems around the GMO detection in Mexico during the last 15 years. The authors highlight the methodology and sampling aspects that could be improved or established in order to get comparable and reliable results, even if they missed a critical point: metrology.

This is an example of a scientific problem where there are different results for supposedly the same measurements. All scientific efforts bring knowledge and contribute to understanding the complexity of the measurable quantity they are investigating. Here, the quantity is the transgene presence or absence and if that is the case, it is necessary to carry out a quantification. Additionally, the quality of measurements must be established in order to perform comparable and reliable analyses (Félix‐Urquídez et al., 2015; Matsuoka et al., 2002). On the other hand, it is crucial to guarantee the traceability of the measurements to the International System of Units (SI) by an uninterrupted chain of comparisons from the national standards or reference materials to the final measurements in the laboratory.

Misunderstandings related to a lack of measurement quality could result in false conclusions. Metrology science establishes the grounds to support measurements through primary standards, reference materials, and uncertainty estimation. In chemical and biological measurements, in general, reference materials are the practical solution for traceability, reliable, and comparable measurements.

## 2 REFERENCE MATERIALS

### 2.1 Metrology

Metrology is the science of measurements in physic, chemistry, biology, etc. Laboratories that develop basic science or technology need a deep knowledge of the measurement process, in order to identify and quantify all the factors that affect the results. National Metrology Institutes establish the standards for the basic units in the International System of Units (SI) for mass, temperature, amount of substance, luminous intensity, length, time, and electricity (JCGM, 2008).

For measurements in the chemical and biological areas, primary standards are used to measure some quantities directly, as in the case of pH and conductivity. Alternatively, the quantity could be described using a fundamental law. In general, chemical or biological measurements are not direct; a measurement result is obtained from the response of an instrument in terms of counts or intensity that we relate with a concentration and with the mol. Thus, it is necessary to develop practical tools to quantify and assure the quality of measurements in this field, which we call reference materials.

National Metrology Institutes (NMI) verify their capabilities through constant participation in key comparisons, establishing a Key Comparison Reference Value (KCRV; Metrologia, 2018). Key comparisons organized by the Consultative Committee for Amount of Substance: Metrology in Chemistry and Biology (CCQM), a consulting organ of the International Committee for Weights and Measures (CIPM), demonstrate calibration and measurement capabilities and comparability between National Metrology Institutes (NMI) around the world. Comparison results and NMI capabilities are publicly available in the Key Comparison Data Base at https://kcdb.bipm.org.

Specifically to GMO detection, it is important to establish which is the certified measurand and its uncertainty, for example, if it is an event or a screening marker p35S or t‐NOS, because, depending on the event, these markers can appear once or twice in the same event. For example, t‐NOS appears twice in the NK‐603 event, and this can result in a wrong interpretation if NK‐603 RM is used to evaluate the presence of GMO base on t‐NOS concentration.

There are different sources of errors that can influence the result of quantification of GMO, which can be classified as type I and II errors. In addition, contamination by the laboratory can occur during sample handling, but this could be controlled by a traceability plan, which must begin from the moment of sampling through the correct storage before, during and after its analysis. Another source of error that should be considered is the adventitious presence of GM sequences, which is closely related to the proper management of the sample. Experimental determination of the adventitious presence of GM material in non‐GM maize fields is usually carried out by applying validated event‐specific GM quantification methods, mainly qPCR to grain samples taken at different locations within the field of interest.

### 2.2 Maize reference materials

According to the International Vocabulary of Metrology, VIM, a Reference Material is a: “material, sufficiently homogeneous and stable with reference to specified properties, which has been established to be fit for its intended use in measurement or in the examination of nominal properties” (JCGM, 2008).

The National Center of Metrology (CENAM) in Mexico started a project related to the development of a Certified Reference Material (CRM) for GMO specific events and *p35S* and t‐NOS, measurands, among others traditionally analyzed to screen for transgenic material in foods and the corresponding reference genes like the adh1 (coding for alcohol dehydrogenase 1) gene. The project began in 2008, and after 10 years, equipment and methodologies have been implemented to carry out the development and certification of CRM in this area.

The equipment with the technology of droplet digital coupled with polymerase chain reaction (ddPCR) is considered a primary method, because it is capable of producing an absolute measurement (Félix‐Urquídez et al., 2015; Pérez‐Urquiza & Acatzi, 2014; Rebecca et al., 2011). Methods like real‐time PCR (qRT) need a calibration curve that increases the measurement uncertainty. Because CENAM is the National Metrology Institute, it is its duty to establish the standards of measurement with the best technology and capabilities to provide the traceability needed.

CENAM's biometrology group certified in one project 6 CRM for soy MON04032 and dry‐resistant wheat, and in a second project 9 additional CRM, 3 for maize MON810, 3 for soy MON04032, and 3 for dry‐resistant wheat. Each material was prepared with genetically modified granulated maize, wheat, and soy donated by a commercial company. The RM were prepared following the CENAM's protocols, developed following ISO 17034:2016, ISO Guide 35:02017 and ISO 21570:2005(E), (ISO, 2005, 2016, 2017) . In general terms, the granulated raw material was milled, sieve and dried in order to obtain a particle size distribution between 150 µm and 425 µm, then distributed in glass bottles with a nominal weight of 1 g. The certified GMO content was from 1% to 98%. To avoid cross‐contamination were followed a strict preparation protocol. All the process was carried out in clean rooms using additionally safety clean benches class II B3.

The homogeneity and stability for each batch were evaluated, and the data obtained were statistically evaluated though an analysis of variance (ANOVA) for each gene of interest (ISO, 2016). Real‐time PCR and digital droplet PCR were used for the certification process. For each CRM, the uncertainty value corresponding to the certified value was estimated as *U* = *ku*_c_, where *u*_c_ is the combined standard uncertainty evaluated according to the Guide to Expression of Uncertainty in Measurements (JCGM 100:2008, 2010).

Additionally, one CRM for cross‐contamination, soy flour/Maize flour (Lec1/hmgA) (identification code DMR 552) and a maize GMO negative control, code DMR 482a were certified by ddPCR. The raw material is supplied by the International Center for Maize and Wheat improvement (CIMMyT), and the CaMV p35S/*hmgA* (cp/cp%) and t‐NOS/hmgA genes were not detected. Additionally, CENAM developed a series of RM for detection and quantification of GMO events, p35S/*hmgA* and t‐NOS/*hmgA*.

The methodologies used to certify the maize RM were validated to obtain reliable and comparable results by cdPCR and ddPCR (Félix‐Urquídez et al., 2015; Metrologia, 2018; Pérez‐Urquiza & Acatzi, 2014).

### 2.3 The use of reference materials

Reference materials can be used for different purposes, such as a method validation technique, quality control in the measurement process, to obtain traceability, calibrate an instrument and to quantify a measurand by a calibration curve.

Validation using RM helps laboratories to establish detection and quantification limits, working interval, interferences, uncertainty, and robustness. Additionally, if the methods used by the laboratory are normalized, they need to be verified when they are implemented in the laboratory. Laboratory capabilities can be evaluated and improved using reference materials, especially when a laboratory begins working on a specific measurement.

The CRMs are available for any laboratory though National Metrology Institutes and commercial producers. An extensive catalog for all the CRM around the World could be found in the following link, http://www.comar.bam.de.

## 3 CONCLUSION

The Mexican Government is working on encouraging the production of more scientific information related to maize native species, though the characterization of Mexican diversity. The public policy improvement or modification will be based on information provided for the scientific data. National Metrology Institutes as CENAM have produced and certified CRM for the identification and quantification of GMO. These CRMs certified under the MRA from CIPM are equivalent and recognized all around the world by signatories. Laboratories can use these CRM for quality control and method validation. Using these practical tools will make it possible currently and for future measurements of GMO to produce comparable results among different research groups that evaluate GMO dispersions in Mexican maize varieties. Additionally, these CRM can provide information on the performance of the equipment and the methodologies. Uncertainty information will be very valuable to assess the results between different methodologies and equipment and to solve controversies due to conflicting results.

## CONFLICT OF INTEREST

The authors have identified no potential conflict of interests.

## AUTHOR CONTRIBUTIONS

Esther Castro conceived the ideas and received some additional ideas from co‐authors during the contrast of the entire article. Esther Castro, Melina Pérez, and Mauricio Maldonado contrasting the whole paper. Esther Castro led the writing. Melina Pérez drafted the review.

## Data Availability

Authors agree to deposit the data to a public repository.
